# Fistulo‐Jejunostomy for Bilio‐Cutaneous Fistula After Hydatid Cyst Surgery: A Case Report

**DOI:** 10.1155/cris/3932089

**Published:** 2026-10-30

**Authors:** Kenan Mohsen Sbh, Abdul Ghani Alshalabi

**Affiliations:** ^1^ Department of General Surgery National University Hospital Faculty of Medicine Damascus University Damascus Syria damascusuniversity.edu.sy; ^2^ Department of General Surgery Homs University Hospital Faculty of Medicine Homs University Homs Syria; ^3^ Department of General Surgery Professor of General and Laparoscopic Surgery Faculty of Medicine Damascus University Damascus Syria damascusuniversity.edu.sy

**Keywords:** bilio-cutaneous fistula, case report, cysto-biliary communication, fistulo-jejunostomy, hydatid cyst

## Abstract

Echinococcosis is a common disease in the Mediterranean region, frequently affecting livestock breeders and farmers. Hepatic involvement is the most prevalent manifestation, typically managed through surgical intervention, either open or laparoscopic. Communication between the cyst and the biliary ducts may occur at varying rates and can be identified preoperatively or postoperatively. Biliary leakage usually resolves spontaneously; however, in some cases, medical intervention via endoscopy, radiological techniques, or surgery may be required, particularly in complex scenarios. In this paper, we present a case of a 63‐year‐old Syrian woman who underwent multiple surgical procedures and interventions to repair a bilio‐cutaneous fistula following open hydatid cyst surgery. The condition was successfully treated using fistulo‐jejunostomy, with no reported complications over a 36‐month follow‐up period.

## 1. Introduction

*Echinococcus granulosus* is a parasite that infects animals and humans, causing echinococcosis. This parasite has a complex life cycle in multiple hosts: carnivores as the definitive host and mammals, including humans, as intermediate hosts. The adult worms live in the intestine of the definitive host, while the larvae live in the viscera of the intermediate host [1]. The most commonly affected organ in humans is the liver (50%–70%) [2], and the right lobe was affected in 79.09% of cases [3]. The most common complication is cysto‐biliary communication (CBC) [4], with an incidence ranging from 2% to 11% [5].

Gharbi et al. were the first to classify hepatic hydatid cysts based on ultrasound [6]. Later, this classification was modified by the WHO Informal Working Group on Echinococcus by adding the cystic pyogenes stage and reciprocally defining types 2 and 3. Additionally, type 3 was generally defined. In comparison, CL, as a potentially parasitic cyst, and WHO CE3b had not been explicitly described by Gharbi [7]. In 2025, the classification was updated to further divide the disease into active cysts (CE1, CE2, CE3a, and CE3b) and inactive stages (CE4, CE5) [8].

Medical therapy may be used as a definitive treatment in selected patients with appropriate cyst characteristics, but it is particularly important as an adjunct to interventional treatment. Perioperative albendazole is commonly administered in repeated treatment cycles or as continuous therapy for several weeks to months, depending on the clinical setting and treatment strategy. Benzimidazole derivatives can be administered before and after surgical intervention with the objectives of reducing cyst viability, minimizing the risk of secondary implantation, and decreasing the likelihood of recurrence [9].

PAIR (puncture, aspiration, injection of a scolicidal agent, and reaspiration) is an established minimally invasive treatment for selected uncomplicated hepatic hydatid cysts [10]. In combination with albendazole, percutaneous drainage has shown similar outcomes to surgery [11]. The PAIR method is effective in reducing biliary fistula development [12]. However, PAIR is not universally applicable. Surgery remains the preferred treatment option for complex cases such as complicated, large, symptomatic, infected, multiple cysts, or biliary fistulas [13].

The surgical approach may be broadly classified as conservative or radical. Conservative procedures include cyst evacuation, partial pericystectomy, or capsulorrhaphy followed by management of the residual cavity, with or without partial pericystectomy, depending on the operative findings. Radical procedures include total pericystectomy and anatomical liver resection [14].

A “missed cyst” refers to a hydatid cyst that remains untreated after the initial intervention and is subsequently identified during follow‐up or a later operation. Inactive, completely calcified, asymptomatic cysts may be managed conservatively with clinical and radiological surveillance when there is no evidence of infection, mass effect, or other complications. In contrast, viable or complicated residual cysts may require medical, percutaneous, or surgical treatment.

The residual hepatic cavity is an additional challenge following surgical evacuation. An inadequately managed residual cavity may become a potential site for bile leakage, secondary infection, hemorrhage, or persistent communication with the biliary tree. Several techniques have been described for managing the residual cavity, including external drainage, capitonnage [15], and omentoplasty [5].

CBC is among the most clinically important complications of hepatic hydatid disease [4]. This complication may manifest as obstructive jaundice, cholangitis, biliary colic, hydatid material within the common bile duct, or persistent postoperative external biliary drainage as a bilio‐cutaneous fistula [4, 14].

The cyst ruptures into the large or small bile ducts in 80%–90% of cases; however, the clinical presentation varies, with estimated rates ranging from 1% to 17% and 13% to 37% [4, 16, 17].

CBCs are classified into two types. A “major” (frank) rupture involves the direct drainage of cyst contents into a major bile duct, causing intermittent or complete ductal obstruction in about 5%–17% of patients. A “minor” (occult or simple) rupture is typically asymptomatic but may lead to a postoperative bilio‐cutaneous fistula in 13%–37% of patients [4].

Bilio‐cutaneous fistula may be visually apparent after excision of the cyst wall, or the condition may manifest as a bile collection in the drain or may develop and present with abdominal wall swelling and tenderness or the formation of a biloma.

Several studies have identified a history of cholangitis, elevated bilirubin and alkaline phosphatase levels, a cyst diameter exceeding 10 cm, and ultrasound findings suggestive of intracystic cholangitis as clinical predictors.

ERCP has been proposed to determine the presence of communication between the cyst and the bile duct in such cases. However, ERCP is the first line to treat bilio‐cutaneous fistula after surgery in case of failure of the conservative treatment [18]. Surgical management is essential for treating complicated lesions, including obliteration of the cystic cavity, repair of the bile ducts, or good drainage [19].

CBCs pose a significant therapeutic challenge, including bilateral bipolar drainage, debridement of the cyst and bile ducts, internal drainage through the fistula, direct suturing of the fistula, and liver resection [20]. Furthermore, it is noted that primary suture repair carries a high failure rate with a success rate of only 29%, whereas radical surgery is more successful [21]

This case report has been presented in line with the SCARE Checklist [22].

## 2. Case Report

A 63‐year‐old female smoker working as a farmer with a body mass index (BMI) of 36 and a medical history of hypertension and type 2 diabetes was diagnosed with hepatic hydatid disease 12 years ago. In 2012, an open surgery (cystotomy and evacuation of the cyst contents) was performed, and the surgery (cystotomy, evacuation, and omentoplasty) was repeated a year later due to the presence of residual cysts. After the second operation, bile oozing occurred at a rate of 200 mL per day. After 1 month of unsuccessful monitoring, ERCP was performed with endoscopic sphincterotomy, which demonstrated normal extrahepatic biliary ducts with communication between the right intrahepatic biliary ducts and the cavity of a large hepatic hydatid cyst. A 5‐cm, 7‐Fr plastic biliary stent was placed to facilitate biliary drainage. After that, stent migration occurred on POD 30. The daily drain output gradually decreased to less than 100 mL per day.

ERCP was repeated several times as described in Table 1, as the oozing continued until the patient underwent another open surgery to repair the bilio‐cutaneous fistula in 2020. In this procedure, the fistula tract was sutured, and omentoplasty and drainage were performed. The drainage decreased to less than 10 mL per day, and the drain was removed on day 25 with the continued oozing of foul‐smelling, yellowish–green fluid.

**Table 1:** Clinical timeline.

Date	Procedure and description	Notes and clinical manifestations	ELISA
2012	First open surgery (cystotomy and evacuation) 3 large hydatid cysts in the right lobe of the liver, drain was removed POD 14	Recurrence of right hypochondrial pain 1 year later	+
2013	Second open surgery (cystotomy, evacuation, omentoplasty, and drainage) During the operation no biliary leakage was observed	The drain spontaneously dislodged on POD 20. Bilious drainage was 200 mL per day	+
2013	First ERCP (sphincterotomy and stenting)	Stent migration on POD 30. Bilious drainage less than 100 mL per day	−
2014	Second ERCP (previously performed sphincterotomy), restenting	Bilious drainage 20 mL per day	−
2014	Third ERCP (same as before)	Bilious drainage 5–10 mL per day	−
2020	Third open surgery to repair the fistula (fistula excision and suturing, omentoplasty, and drainage)	The drainage decreased to less than 10 mL per day, and the drain was removed on POD 25, with persistent oozing of a foul‐smelling, yellowish–green fluid	−
2023	Fourth ERCP (wide endoscopic sphincterotomy restenting 7‐Fr plastic biliary stent with two pigtail ends)	The biliary stent was left for 60 days before, and 30 days jejunostomy	−
2023	End‐to‐side fistulo‐jejunostomy in an omega configuration	Drain removed on POD 7	−
2023–2026	Follow‐up	Cure	

*Note:* +, positive; −, negative.

Abbreviations: ERCP, endoscopic retrograde cholangiopancreatography; POD, postoperative day.

Regarding the history of treatment with benzimidazoles (15 mg/kg/day in two divided doses), she underwent a 6‐month course of treatment in 2012 and another course of treatment after the second operation in 2013, and then she received multiple courses of albendazole despite negative serological tests.

In 2023, the woman was presented to the surgery department with daily discharges of no more than 10 mL of viscous, yellow–green, foul‐smelling purulent fluid from the drain site, which had a 2 mm fistula orifice.

Clinical examination showed mild tenderness in the right hypochondrium without fever or jaundice and no limitation in function.

Upon admission, the blood pressure was 150/90 mmHg, pulse was 80/min, and oxygen saturation was 96%. Laboratory investigations were within normal limits except for a decrease in hemoglobin to 9.4 g/dL and high‐glucose C‐reactive protein of 90 mg/L. Liver enzymes were normal, and there was no increase in alkaline phosphatase; also, the serological tests for *Echinococcus granulosus* were negative (Table 2).

**Table 2:** The laboratory tests upon admission.

Test	Value	Test	Value
WBC	5.9 × 109/L	AST	9 U/L
Neutrophil	63%	ALT	11 U/L
Hemoglobin	9.4 g/dL	Total bilirubin	0.25 mg/dL
Hematocrit	29%	ALP	102 U/L
PLT	217 × 109/L	Na	138 mmol/L
INR	1	K	4.6 mmol/L
Urea	42 mg/dL	Ca	9.2 mg/dL
Creatinine	1.3 mg/dL	TSH	1.11 µUI/m
TP	7.4 g/dL	Glucose	167 mg/dL
Alb	4.1 g/dL	CRP	90 mg/L
ELISA	Negative	IHA	Negative

Abbreviations: ELISA, enzyme‐linked immunosorbent assay; IHA, indirect hemagglutination.

A pus sample was collected directly from the cutaneous fistula opening and sent for microbiological culture and antibiotic susceptibility testing. It revealed the presence of *Staphylococcus aureus* bacteria. Linezolid was given intravenously during the preparation period for the surgical procedure. During surgery, a piece of the fistula wall was cultured, showing no bacterial growth after 72 h. After surgery, intravenous antibiotics were continued for a week, then orally for another week until the wound has completely healed.

An abdominal ultrasound revealed a mixed‐echoic hepatic cyst without biliary dilatation, and the common duct measured 5 mm.

A multislice computed tomography (CT) fistulography was performed following direct injection of contrast material through the cutaneous fistula orifice. It opacified three interconnected cavities and subsequently passed through the biliary tree, reaching the second portion of the duodenum. The extrahepatic biliary ducts were normally opacified (Image not available).

A multislice contrast‐enhanced CT scan showing three interconnected, calcified cystic cavities in segments 3–4 (Figure 1), with a 3D reconstruction illustrating their spatial relationship (Figure 2).

**Figure 1:**
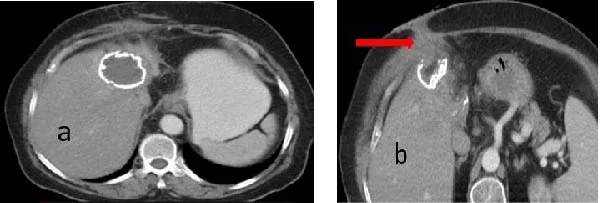
Contrast‐enhanced computed tomography (CT) images demonstrate a complex hydatid cyst with a calcified wall located in liver segments III and IV. (a) Axial images show a cyst with peripheral calcification, measuring 5 cm in diameter and having a wall thickness of 0.5 cm. (b) A fistula connecting the skin to the liver surface, which does not enhance with contrast (red arrow) because the CT scan was not synchronized with the injection into the fistula.

**Figure 2:**
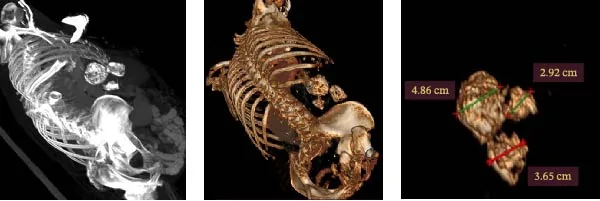
Contrast‐enhanced CT scan showing three interconnected, calcified cystic cavities in segments III and IV. 4.86 cm, 3.65 cm, and 2.92 cm in diameter. A 3D reconstruction demonstrating their spatial relationship.

ERCP in 2023 revealed a normal papilla. Cholangiography demonstrated normal intrahepatic and extrehepatic biliary ducts. A wide sphincterotomy was performed, and a 7‐Fr plastic biliary stent with two pigtail ends was performed. Despite this, after 2 months, the oozing from the fistula did not improve.

## 3. Description of the Surgical Procedure

A right subcostal Kocher incision was made. Access to the abdomen was established after peritoneal and muscle dissection. Dense adhesions were found, with the right colon wall adhering to the internal peritoneum and liver. The omentum was strongly adhered to the liver surface and embedded in the precyst cavity. The cyst cavity, filled with omentum, was estimated to be ~4–5 cm in diameter. No purulent collections were observed. The fistula, extending from the skin to the liver surface, was estimated to be over 10 cm long. Due to the severe adhesions and bleeding that occurred during dissection and adhesion release, it was decided to isolate the fistula tract. This was achieved by separating it from the skin and adipose tissue and extending it to the liver surface as a channel. After isolating the fistula tract and ensuring its integrity using saline and methylene blue, the edges were debrided. Figure 3 shows the fistula orifice after debridement. The internal diameter of the fistula was ~0.5 cm, with an approximate wall thickness of 0.5 cm, including the surrounding fat. The internal wall of the fistula also appears as a granulation tissue. A jejunal loop was mobilized, and an omega‐shaped anastomosis was performed. A 0.5‐cm enterotomy was created on the antimesenteric border of the jejunum, ~70 cm distal to the ligament of Treitz. The jejunum was then anastomosed laterally to the distal end of the fistulous tract using a side‐to‐end configuration. The anastomosis was performed with interrupted 2–0 Vicryl sutures in a single layer. After confirming the integrity of the anastomosis, the area was irrigated with a saline solution, and a corrugated drain was placed next to the anastomosis.

**Figure 3:**
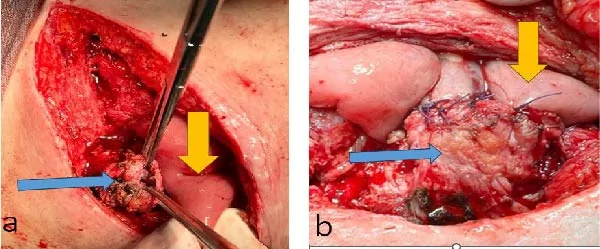
Intraoperative image of fistulo‐jejunostomy: (a) preparing the anastomosis, the internal diameter of the fistula was 0.5 cm, with 0.5 cm wall thickness. (b) interrupted, single‐layer 2–0 vicryl anastomosis (complete fistulo‐jejunostomy). Jejunum limp (yellow arrow), isolated mature fistula (blue arrow).

The patient was monitored in the surgical unit for a week without any complications (Clavien–Dindo grade 0). The drain was removed in POD 7, and the patient was discharged. She was routinely followed up. Over the 36‐month postoperative surveillance period, the patient remained clinically well, with a stable BMI and no reported episodes of abdominal pain or jaundice. Serial liver function tests, including transaminases and alkaline phosphatase, remained within normal limits. Repeated ultrasound examinations performed throughout this period showed no evidence of biliary obstruction. Three years have passed since the surgery, and the patient has not complained of any complications or recurrence of purulent discharge.

## 4. Discussion

Hepatic hydatid disease, predominantly caused by the larval stage of *Echinococcus granulosus*, the liver is the most frequently involved organ, accounting for the majority of cases [3].

The clinical presentation and management of hepatic hydatid cysts are highly variable and depend on several factors, including cyst stage, size, anatomical location, relationship to the biliary tree, and the presence of complications.

Management should therefore be individualized within a multidisciplinary framework. Current therapeutic options include medical therapy with benzimidazole derivatives, minimally invasive percutaneous procedures, and surgical intervention. The choice of treatment is determined not only by cyst size but also by its biological activity, structural characteristics, biliary communication, and the presence of complications such as infection, rupture, or cysto‐biliary fistulization [8, 23].

Hepatic cysts can rupture into the bile duct, which can lead to life‐threatening complications. The postoperative biliary fistula after liver hydatid cyst surgery is a common complication, with an incidence of 5%–25% [24].

Small, low‐output biliary fistulas may close spontaneously, particularly when adequate distal biliary drainage is maintained and there is no persistent obstruction or uncontrolled infection, while large leaks are resistant to endoscopic or interventional treatment [25].

ERCP is effective for persistent or clinically significant cysto‐biliary fistulas. Endoscopic sphincterotomy, with or without stent placement, can reduce the pressure gradient across the sphincter of Oddi and preferentially divert bile flow into the duodenum rather than through the fistulous tract [3]. It also offers an additional benefit: providing useful anatomical information for selecting the best treatment approach [21]. Inadequate decompression may lead to chronic bile leakage into the cyst cavity with persistent external bile drainage or even suppuration of the residual cavity [5, 26].

A 2025 study involving 88 patients, with leakage occurring in 82% of cases following surgery and 16% following PAIR demonstrated the efficacy and safety of ERCP in managing biliary leakage after surgery or PAIR; the procedure achieved an 83% complete closure rate within 20 days using stents [27].

Surgical intervention is reserved for selected patients with persistent fistulas that fail to respond to adequate conservative and endoscopic management.

Direct surgical repair may involve identification of the biliary communication, closure of the fistulous opening, management of the residual hepatic cavity, and biliary drainage according to the intraoperative findings [28, 29].

In selected patients with chronic or recurrent fistulization in whom direct repair is unsafe or technically impossible, a biliodigestive anastomosis as a Roux‐en‐Y hepaticojejunostomy may provide an effective method of establishing a low‐pressure biliary drainage pathway [30]. A Roux‐en‐Y‐type pericystojejunostomy may be preferred in large fistulas opened to the main biliary tract or if the cavity is large [23]. In cases where reconstruction is difficult, recourse may be had to radical surgery involving pericystectomy or resection [5, 31]. This method carries potential risks, including bleeding, infection, anastomotic leakage or stricture, and recurrent cholangitis.

Medical literature describes some unusual cases of bilio‐cutaneous fistula closure as pericystogastrostomy [32], Roux‐en‐Y cystojejunostomy [33], N‐butyl 2‐cyanoacrylate embolization [34], nasobiliary tube [35], percutaneous cyanoacrylate injection [36].

Consequently, we adopted a less invasive, older technique in a complex reoperative setting previously successful in managing biliary or digestive fistulas. Given the history of multiple interventions on the cysts, the presence of adhesions and prior omentoplasty, low fistulous output, and a well‐formed mature fistula tract, we elected to perform a direct anastomosis between the fistula and the digestive tract. Fistulo‐jejunostomy is an unusual procedure for treating chronic, refractory fistulas. It was first described in 1962 [37]. Its use has declined with the development of endoscopic and interventional radiology techniques [38]. Reports have documented the success of the procedure in numerous cases of biliary fistula following hepatectomy and cholecystectomy, biliary‐jejunal anastomosis leakage following the Whipple procedure, and trauma [38–40] with a success rate as high as 70% [40].

Currently, fistulo‐jejunostomy is performed for fistulas after refractory external pancreatic fistula [41], gastric resection [42], refractory esophagogastrostomy [43], and other gastrointestinal fistulas, with good results.

The fistula must be mature and chronic. The term “mature” is not used in a purely chronological context. Rather, it refers to a fistula that has become formed and stable, meaning that the connection between the bile ducts and the skin becomes continuous through the cyst cavity, with a defined pathway lined by epithelium or granulation tissue, and that bile continues to drain through the skin duct at a steady and regular rate. Clinically, it is important to assess whether the fistula is on the path to spontaneous closure or will become persistent or mature. This can be predicted by a decrease in bile flow, the absence of a collection or abscess around the liver, the absence of biliary obstruction, and, most importantly, the complete cessation of bile flow without internal accumulations. Generally, based on the literature, the average closure time was ~30 days, which may be an indicator of maturity [14]. In a study of 46 patients after hydatid cyst surgery, the average spontaneous closure was about 10 days, while in cases requiring biliary intervention, the average was 31 days [14, 21]. In another study, median time of spontaneous closure is reported between 4 and 36 weeks [40] or 3 months in another report [43]. Therefore, it can be said that after this timeframe, and with continued and steady leakage, the fistula has become more mature and often requires surgical intervention.

In other literature studies, it was considered that a flow rate of more than 300 mL/day is considered high and requires ERCP if it continues for more than a week, while it is possible to wait up to 3 weeks in fistulas with low flow rates of less than 300 mL/day [44]. When inadequate maturity of the fistula wall is perceived at surgery, the fibrotic liver capsule, around the fistula opening, can be sutured to the jejunal wall, and the anastomosis may be reinforced with an omental flap or stented with a drainage tube brought out through the jejunal wall [43, 45].

Given the presence of severe adhesions and the difficulty of surgical exploration, compounded by the patient’s obesity (BMI = 36), a history of infection and perihepatic inflammation, and multiple prior failed interventions (including omentoplasty) [40], and in the absence of a specialized liver surgeon to perform hepatectomy, we determined that such a procedure would entail significant risks, including potential hemorrhage or injury to the liver and adjacent organs. Therefore, the fistulo‐jejunostomy in this setting can therefore be viewed as a form of biliary diversion or bypass rather than simple fistula closure. By providing a low‐resistance pathway for bile drainage, the procedure reduces the physiological driving force for bile flow through the abnormal cutaneous tract and may facilitate its eventual functional closure.

This case emphasizes an important principle in complex reoperative hepatobiliary surgery: the safest operation is not necessarily the most anatomically aggressive operation. When the fistulous tract is mature, the surrounding anatomy is severely distorted, adhesiolysis carries a high risk of major hemorrhage, and fistulo‐jejunostomy may represent a rational and safer alternative to extensive dissection and direct fistula excision.

## 5. Limitations

This case report has inherent limitations, including its single‐patient nature and the lack of high‐resolution preoperative CT imaging for publication, the loss of the ERCP images due to the war and forced displacement, and the loss of the archives of the health center where the procedure took place.

## 6. Conclusion

Hepatic hydatid disease requires individualized management based on cyst characteristics, disease stage, anatomical relationships, complications, and the patient’s overall condition. Although most cystobiliary fistulas can be managed successfully with conservative or endoscopic approaches, chronic and refractory fistulas following previous hepatic surgery may present formidable technical challenges.

In patients with a mature fistulous tract and a hostile operative field characterized by dense vascular adhesions, aggressive adhesiolysis and direct fistula excision may carry disproportionate morbidity. In such carefully selected circumstances, Fistulo‐jejunostomy can provide an effective alternative by establishing low‐pressure biliary drainage and bypassing the fistulous pathway without requiring extensive dissection of the adherent liver.

The present case therefore highlights the importance of individualized, risk‐adapted surgical decision‐making in complex recurrent hepatobiliary disease.

## Author Contributions

Kenan Mohsen Sbh designed, performed the surgical procedure as the primary surgeon and supervised postoperative care, conceptualized the initial idea for the case report, acquired the patient’s clinical and laboratory data, wrote the original draft of the manuscript, and participated in revising the manuscript and incorporating reviewers’ feedback. Abdul Ghani Alshalabi supervised the operative and approved the final manuscript.

## Acknowledgments

No artificial intelligence tools were used in the manuscript preparation for this case report.

## Funding

The authors received no specific funding for this work.

## Ethics Statement

The authors have nothing to report.

## Consent

A written informed consent was obtained from the patient for publication and any accompanying images. A copy of the written consent is available for review by the Editor‐in‐Chief of this journal on request. The institutional ethics committee approved the publication.

## Conflicts of Interest

The authors declare no conflicts of interest.

## Data Availability

The data are available from the corresponding author upon reasonable request, subject to institutional and patient‐consent constraints.
